# Recent Progress in Creep-Resistant Aluminum Alloys for Diesel Engine Applications: A Review

**DOI:** 10.3390/ma17133076

**Published:** 2024-06-22

**Authors:** Raul Irving Arriaga-Benitez, Mihriban Pekguleryuz

**Affiliations:** Department of Mining and Materials Engineering, McGill University, 3610 University, Montreal, QC H3A 0C5, Canada; mihriban.pekguleryuz@mcgill.ca

**Keywords:** second phase, Al alloy, creep, heavy vehicle, diesel engine component

## Abstract

Diesel engines in heavy-duty vehicles are predicted to maintain a stable presence in the future due to the difficulty of electrifying heavy trucks, mine equipment, and railway cars. This trend encourages the effort to develop new aluminum alloy systems with improved performance at diesel engine conditions of elevated temperature and stress combinations to reduce vehicle weight and, consequently, CO_2_ emissions. Aluminum alloys need to provide adequate creep resistance at ~300 °C and room-temperature tensile properties better than the current commercial aluminum alloys used for powertrain applications. The studies for improving creep resistance for aluminum casting alloys indicate that their high-temperature stability depends on the formation of high-density uniform dispersoids with low solid solubility and low diffusivity in aluminum. This review summarizes three generations of diesel engine aluminum alloys and focuses on recent work on the third-generation dispersoid-strengthened alloys. Additionally, new trends in developing creep resistance through the development of alloy systems other than Al-Si-based alloys, the optimization of manufacturing processes, and the use of thermal barrier coatings and composites are discussed. New progress on concepts regarding the thermal stability of rapidly solidified and nano-structured alloys and on creep-resistant alloy design via machine learning-based algorithms is also presented.

## 1. Introduction

The report presented in 2023 by the International Energy Agency revealed that 10 million electric passenger light-duty vehicles, including cars of different sizes, sports utility-vehicles, and light trucks, were sold in 2022, compared to only 120,000 electric heavy-duty vehicles, including electric buses and medium- and heavy-duty trucks, representing a difference of more than 8000% [1]. While electric cars are making headway in the environmental sustainability of the transport industry, the limited electrification in construction, military, agriculture, mining, and railroad sectors due to challenges in battery technology and charging infrastructure necessitates the implementation of new technologies for heavy-duty vehicles. Lightweighting of diesel engine components through aluminum (Al) substitution is positioned to offer fuel economy, better performance, and increased environmental benefits in heavy vehicles [2,3].

This review is structured into five sections. After a brief introduction in Section 1, Section 2 discusses the importance of the diesel engine for heavy vehicles and summarizes the operating conditions, failure modes, and the early development of aluminum alloys for the diesel engine (first- and second-generation alloys), and Section 3 presents a review of the third generation of alloys. Section 4 focuses on new approaches (materials and processes) that can improve the elevated-temperature performance of aluminum and enable the use of aluminum in the diesel engine. Section 5 provide a partial comparison of the experimental alloys and the future direction in aluminum research for the diesel engine.

## 2. Diesel Engines in Heavy Vehicles, Operating Conditions, Failure Modes, and the Early Development of Aluminum Alloys for the Diesel Engine

In terms of transportation, battery electric vehicles (BEVs) offer highly viable solutions for efficiency, reduction in greenhouse gas (GHG) emissions, and lower operating costs (because they have fewer moving parts). Despite these benefits, the limitations of battery technology, such as poor gravimetric and volumetric energy density, are the main barriers to the electrification of heavy vehicles [4]. Additionally, development in the charging infrastructure is required to scale up the adoption of heavy EVs [5]. A study on army power and energy needs through 2035 [6] indicated that all-battery electric ground combat vehicles are impractical now or in the foreseeable future because of the extensive use of batteries in tactical vehicles, limited use in a battlefield environment, long recharging times, and the need for sizeable electrical power sources. In fact, an analysis on the average global battery capacity in medium- and heavy-duty vehicles from 2019 to 2022 shows that the electric heavy-duty trucks have the largest battery capacity, with a total of 350 kWh [7]. However, this amount of energy is not enough to power heavier vehicles; for example, M109A7 Howitzer (448 kWh), Caterpillar 797F (2983 kWh), freight rail locomotive (3355 kWh), and front loaders, bulldozers, and other construction vehicles use diesel engines to carry loads of heavy materials. Therefore, for heavy vehicles, diesel engines that can generate high torque and high energy density and that have more extensive refueling infrastructure are more viable than any other type of vehicle technology (i.e., electric and gasoline).

### 2.1. Materials, Operating Conditions, and Failure Mechanisms in Diesel Engine Cylinder Head and Engine Block

**Figure 1:**
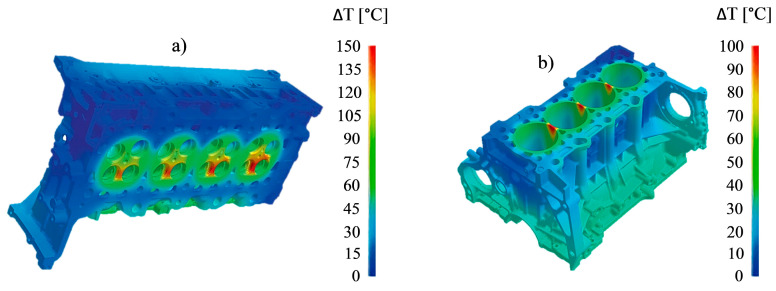
Temperature distribution surfaces at the (**a**) cylinder head and (**b**) engine block for an engine speed of 4000 rpm. Reproduced with permission from [11]. Copyright Springer Nature, 2012.

Historically, cast iron has been used for manufacturing diesel engine components. In cylinder head and engine block production (Figure 1a,b), grey and ductile iron are typically used because of their good castability and machinability, relatively low cost, and good mechanical and thermal properties [8]. Unfortunately, these iron-carbon alloys cannot be used as a choice in vehicle weight design due to their high density. Consequently, switching from cast iron to aluminum alloys would result in very effective weight reduction (40–55%), thereby increasing the environmental benefits, improving the fuel economy, and reinforcing the vehicle performance (i.e., lowering the center of gravity of the engine in a vehicle to improve handling).

The cylinder head and engine block materials are exposed to high compression pressures and temperatures during diesel engine operation. Generally, four types of loads affect the lifetime of these diesel engine components [9]: (i) manufacturing process loads, such as residual stresses due to molding, heat treatment (due to quenching), and machining processes, (ii) assembly loads, such as bolting load and press-fits, (iii) mechanical operating load due to cylinder gas pressure, and (iv) thermal load caused by high in-cylinder gas temperatures and large variation in temperatures during operating cycles. The performance of these components, when constructed in aluminum, is limited by thermal fatigue (also called thermomechanical fatigue) and mechanical fatigue due to the engine start, operate, and stop cycles, as well as repeated combustion events [10]. The thermomechanical cyclic loadings on the cylinder head, namely, in the inter-cylinder bridge zone, are more severe than those in the engine block, as shown in Figure 1.

### 2.2. Mechanical Fatigue in Aluminum Cylinder Head and Engine Block

While this review is on thermal fatigue issues in the cylinder head, a short background on mechanical fatigue in the diesel engine is presented below. In the cylinder head, mechanical fatigue cracks are generated from high peak cylinder pressures or high stresses occurring mostly at the water/cooling ducts (temperature range from 120 °C to 170 °C), where high tensile residual stress occurs during the heat treatment process.

In the engine block, the maximum stress occurs at the engine-block wall and cylinder liner when the lateral force of the cylinder piston generates a maximum explosion pressure. At this point, the cracks are created and expanded under the cyclic effect of the explosion pressure at 120 °C [10]. Other factors include engine geometry, residual stresses, and casting conditions (porosity, secondary dendrite arm spacing, and inter-metallic phase morphology), which can determine the mechanical fatigue performance for both the cylinder head and engine block [12]. Additional loads occurring during the cylinder head and engine block processing can also influence its performance, although they can be reduced or eliminated by using annealing processes [13].

### 2.3. Thermomechanical Fatigue in Cylinder Head and Engine Block

**Figure 2:**
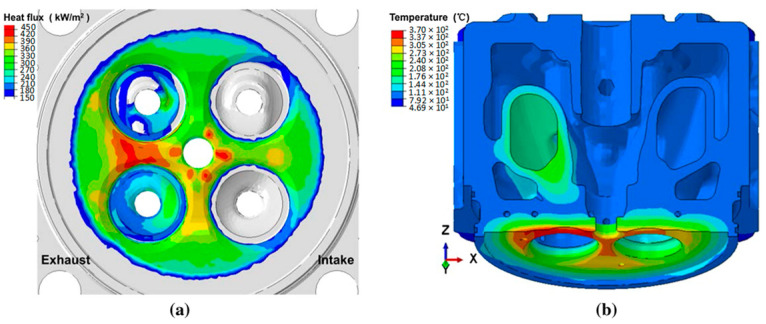
(**a**) Heat flux distribution along the cylinder head fire surface and (**b**) temperature distribution of the cylinder head. Reproduced with permission from [14]. Copyright Springer Nature, 2021.

Thermomechanical fatigue is the most important failure in the aluminum cylinder head. A typical case of cylinder head thermomechanical fatigue is at the inter-valve bridge area, especially between the intake and exhaust valves (Figure 2). As depicted in Figure 2, the maximum heat flux in a marine diesel engine cylinder head is found in the exhaust–exhaust valve bridge due to the exposure to the high-temperature gases during combustion, and it decreases toward the outside of the combustion chamber and cylinder liner (Figure 2a). Similarity, the temperature distribution of the cylinder head under working conditions can reach 368 °C in the bridge area between the two exhaust valves, and it decreases from the cylinder center to the cylinder liner side due to the cooling hole of the water chamber (Figure 2b).

**Figure 3:**
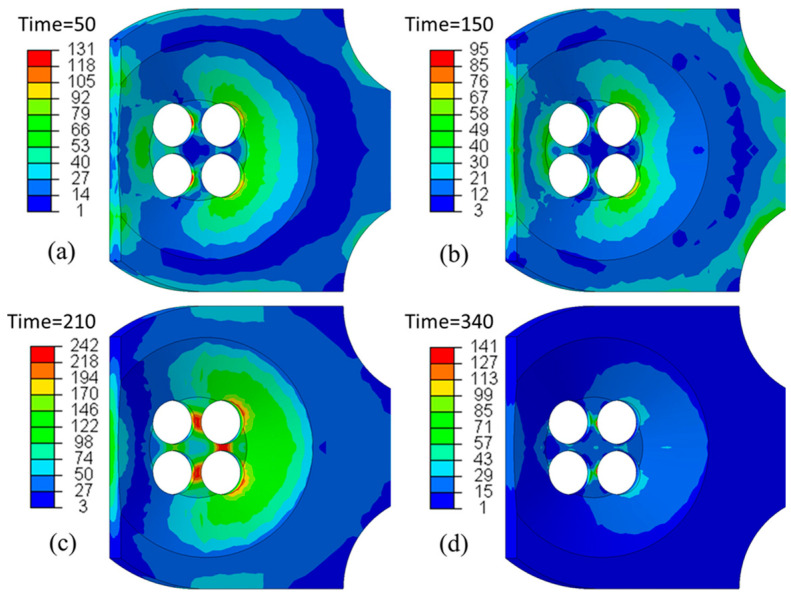
Stress distribution at the typical time during thermal cycling: (**a**) 50 s heating, (**b**) 150 s heating, (**c**) 210 s cooling, and (**d**) 340 s cooling. Reproduced with permission from [15]. Copyright Springer Nature, 2022.

The thermomechanical fatigue is due to the thermal expansion and contraction during engine operation (Figure 3b). During engine heat-up, the inter-valve region reaches 300 °C and the metal tries to expand but is constrained due to the geometry of the region. This puts the metal under compressive loads of around 20–30 MPa. The alloy is subjected to creep deformation due to loading at elevated temperatures. At engine cooldown, the metal tries to contract from its state at elevated temperature, but it is constrained and is placed under tensile loads, leading to tensile deformation, causing the greatest contribution to fatigue at this critical region. The repetition of expansion and contraction cycles identified during engine heat-up and cooldown result in local tensile stresses, causing fatigue cracks that can be nucleated during the engine cooldown period if the initial creep deformation is high and the material does not have sufficient ductility to accommodate these stresses by local plastic deformations. Even when the material is ductile, the repetition of these alternating compressive and tensile stresses can cause crack initiation due to plastic damage accumulation and eventually lead to failure in diesel engines.

Zhang et al. [15] simulated a thermal cycling test of a marine engine cylinder head to evaluate the stress under start–stop cycle conditions. Figure 3 shows the stress distribution of the first cycle. In the heating stage, the local stress concentrates on the exhaust nose bridge area, but at the maximum temperature, a decrease in the stress is seen due to stress relaxation. This stress change is attributed to the material exhibiting cyclic softening with higher temperatures [16]. In the cooling stage, the stress reaches the maximum value, and decreases as time goes on. Consequently, the failure mode of inelastic deformation during the thermal cycle is reproduced by thermal-gradient-cycle-induced structural constraints.

There is no material property that can properly assess the response of the inter-valve region to thermal cycles and stresses during diesel engine operation. Thermal fatigue evaluates the response of the material during thermal cycling when the specimen is totally free to expand or contract so that only mechanical strains resulting from the thermal gradients are experienced by the specimen [17]. Instead, the resistance to the specific type of thermal fatigue experienced by the geometrically constrained inter-valve can be assessed based on two properties of the material: (1) tensile performance at engine cooldown (at room temperature) and (2) creep resistance at engine heat-up (300 °C and 22–30 MPa). For tensile performance, there are two schools of thought that place more importance on either ductility or tensile strength, which depends on whether it is preferable for the material to accommodate the tensile stresses either elastically or plastically during engine cooldown. Therefore, the material property requirements, such as creep resistance at 300 °C to resist creep deformation in the inter-valve regions during engine heat-up, ductility to accommodate and avoid catastrophic failure, and strength to accommodate the tensile stresses elastically when ductility is low, have been the main objectives during the development of diesel engine components based on Al alloys.

### 2.4. The Early Use of Aluminum in the Diesel Engine: First- and Second-Generation Alloys

Over the years, different generations of aluminum diesel engine alloys have been investigated to provide adequate strength and creep resistance under diesel engine operating conditions. A review of first- and second-generation aluminum alloys for use in the diesel engine was conducted by Pekguleryuz et al. [18]. A summary and the principles discovered are provided here.

The first-generation alloys were developed to achieve improved creep resistance as modifications of the commercial aluminum alloys A356 (Al-7Si-0.3Mg-0.2Fe) or the A319 (Al-6Si-3.5Cu-1Fe) used in gasoline engine vehicles. The researchers who valued increasing strength over ductility used modified A319, and those who preferred to maintain ductility used A356 as the base alloy. These alloys are based on the Al-Si system with β′-Mg_2_Si, λ-Al-Mg-Si-Cu, or the θ-Al_2_Cu, Q-Mg-Si-Cu precipitation sequence depending on the Mg and Cu levels, with the second sequence providing higher strength. In the T6 condition, they possess metastable phases that transform and coarsen under diesel engine service temperatures, leaving only the Q precipitate for strengthening.

Aluminum alloy development activities for diesel engines began in 1998 by VAW. These activities focused on adding 0.5–1% copper to the commercial Al-Si casting alloys (319, 356, 380, and 390) to increase strength and creep resistance for use in cylinder head applications [13,19]. Among the alloys optimized, the A356 (Al-Si-Mg) alloy showed the most promising results. Studies indicated that after T6 treatment (solutionizing for 5 h at 525 °C, followed by water quenching and annealing for 4 h at 165 °C), there was no improvement in yield stress or hardness at ambient temperature. However, a significant improvement in yield stress and creep resistance was observed at service temperatures above 150 °C. This behavior was associated with the formation of Cu-precipitates after the T6 heat treatment [18,20,21], but a loss of strength was seen at 300 °C due to the coarsening of the precipitates.

The second generation emerged from the addition of nickel to the A356 + 0.5% Cu alloy for engine block applications, because they require higher strength than the cylinder head [22,23]. The Al-7Si-0.4Mg-0.4Cu-0.3-Mn-0.5Ni-0.4Fe alloy in the T6 condition exhibited creep resistance at 120 MPa and 200 °C, because the time to reach from 0.2 and 0.5% strains was longer than the commercial alloys. In addition to the improvement in creep resistance of 500 h at 250 °C, the fatigue strength also increased by 20%, and tensile strength showed an improvement. This phenomenon was attributed to the formation of Ni-bearing inter-metallics. However, the alloy presented low ductility due to the brittleness of the Ni-bearing inter-metallics.

Some of the points obtained from the studies of the first and second generation of aluminum diesel engine alloys can be summarized as follows:

1. Since peak age T6 is not sustainable in Al alloys for diesel cylinder heads (e.g., time at 300 °C), the nature of the equilibrium precipitate determines the strengthening effect [20]. The strength of the diesel engine alloy needs to be designed on the over-aged/equilibrium-state microstructure, and the tensile properties need to be evaluated after long presoaking at temperature.
2. In the Al-Si-Mg-Cu system, the equilibrium precipitates can vary depending on the Si/Mg ratio and Cu level [18]. This quaternary alloy composition can be adjusted to yield (Q, Si, θ) or (Q, β, Si) equilibrium phases [18]. While the mix of equilibrium precipitates is well known in wrought Al alloys with low Si levels, the precipitate mix versus composition in experimental casting alloys with higher Si is not well documented. The authors suggest that the precipitates in these experimental alloys can be predicted/determined via thermodynamic calculations, scanning calorimetry (DSC) validations, and high-resolution transmission electron microscopy (HRTEM). These can be viable research directions in future studies.
3. Nickel additions to the Al-Si-Mg-Cu alloy usually led to low ductility due to the existence of the brittle T-phase [22,23], which can lead to early failures in tensile or creep testing.

## 3. Review of the Third Generation of Aluminum Diesel Engine Alloys

The development of the third generation of aluminum diesel engine alloys focused on improving the most important performance requirements of creep resistance at 300 °C to resist the deformation in the inter-valve area [18,24]. The alloys were designed to form a high-volume fraction of thermally stable dispersoids with the addition of peritectic elements to Al-Si-Mg-Cu alloy compositions.

### 3.1. Early Work on Third-Generation Aluminum Alloys for the Diesel Engine

**Figure 4:**
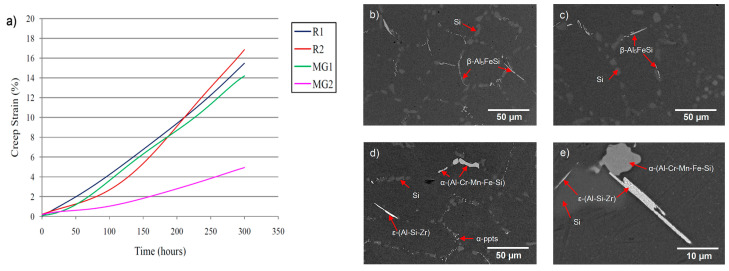
(**a**) Compressive creep curves at 300 °C under 30 MPa and SEM micrographs of the (**b**) R1, (**c**) R2, (**d**) MG1, and (**e**) MG2 alloys in the over-aged condition [26].

In 2008, Prof. Pekguleryuz’s team at McGill, in collaboration with Rio Tinto (London, UK) (then Alcan Aluminum, Montreal, QC, Canada), developed a creep-resistant aluminum alloy from the combined addition of peritectic elements (Cr, Zr, Ti, and Mn) to the A356 + Cu alloy [18,25]. The alloy (MG2) showed better compressive creep resistance by reaching only a creep strain of 4.9% after 300 h at 300 °C. The improvement in the MG2 alloy was up to three times better than the R1 (A356), R2 (A356 + 0.5% Cu), and the MG1 (A356 + 0.15% Mn +0.15% Cr +0.15% Zr) alloys (Figure 4a). Microstructural analysis in the MG2 alloy revealed that the Zr-Si-Al ternary phase, a complex Cr phase (Al-Cr-Mn-Fe-Si), and Zr submicron phases in the grain interior were responsible for enhancing the creep resistance (Figure 4b).

**Figure 5:**
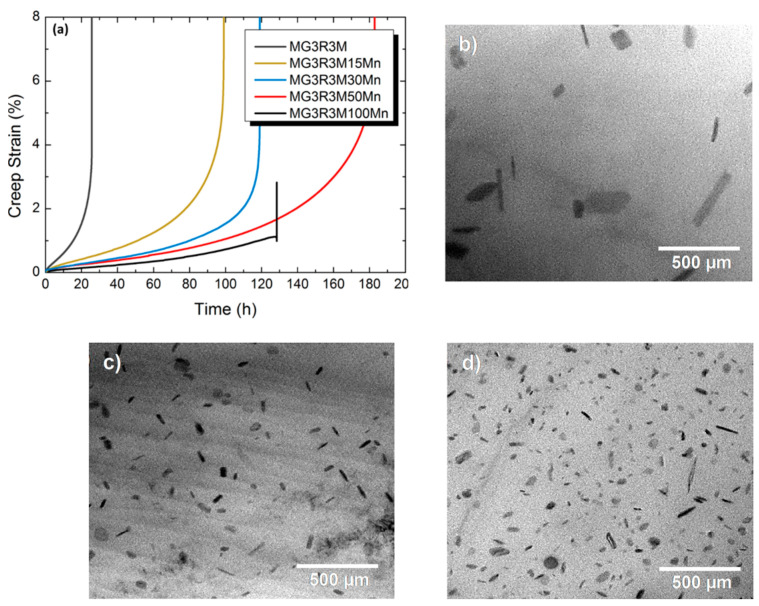
(**a**) Tensile creep deformation vs. time at 30 MPa and 300 °C. TEM micrographs showing the distribution of α-Al(Fe,Mo,Mn)Si dispersoids in (**b**) Al-7Si-0.5Cu-0.3Mg-0.1Fe-0.3Mo, (**c**) Al-7Si-0.5Cu-0.3Mg-0.1Fe-0.3Mo-15Mn, and (**d**) Al-7Si-0.5Cu-0.3Mg-0.1Fe-0.3Mo-0.5Mn at 540 °C after 600 min. Reproduced with permission from [27]. Copyright Elsevier, 2015.

In 2015, Farkoosh and Pekguleryuz [27] demonstrated that molybdenum (Mo) in combination with manganese (Mn) formed a large volume fraction of dispersoids that resulted in the development of highly creep-resistant Al-Si-Cu-Mg-Mo-Mn alloys. Mn was of interest because of its low diffusivity and relatively high solid solubility in the Al matrix. Creep testing results on the Al-7Si-0.5Cu-0.3Mg-0.1Fe-0.3Mo-(0-1.0) Mn alloy at 300 °C and 30 MPa indicated that creep time-to-fracture (Figure 5a) increased from ~25 to ~180 h as Mn increased from 0.0 to 0.5. However, at 1% Mn, creep time-to-fracture dramatically decreased to 130 h. Microstructural observations associated the increase in creep resistance with the increase in the precipitation density of α-Al(Fe,Mn,Mo)Si dispersoids (from ~0.6 to ~4 μm^−2^), reducing the dispersoid-free zone (DFZ; Figure 5d). This pattern resulted from the Mn supersaturation and Mo segregation in the intra-dendritic and inter-dendritic regions, respectively, improving the creep resistance in the Al-7Si-0.5Cu-0.3Mg-0.1Fe-0.3Mo-0.5Mn (MG3RM50Mn) alloy system. It was suggested that Mn, a peritectic element, enriches in the α-Al dendrite cores (as a solid solution) and diffuses slowly, forming the α-Al(Fe,Mn,Mo)Si dispersoids and coarsening slowly, maintaining thermal stability. The decrease in creep resistance at 1 wt% Mn was due to the increased amount of the inter-metallics, which embrittled the alloy. As a result, the dislocation pile-up at the inter-metallic/matrix interface can generate sufficiently high stresses that can cause fracture.

In 2020, Rashno et al. [28] compared the creep behavior of the cast A356 alloy with combined and singular additions of Zr and Er and obtained improved creep resistance due to an increase in the amount of Zr- and Er-rich inter-metallics, characterized by their various shapes and sizes. Fe-rich inter-metallics did not form because Fe atoms were replaced by Zr and Er atoms. As a result, the modified alloy exhibited uniform elongation. Microstructural features also showed that the formation of fine dendritic α-Al and fibrous and partially rounded eutectic Si improved the creep resistance, restricting the mean free path for dislocation motion. Researchers indicated that lattice self-diffusion, climb-controlled creep is the dominant creep mechanism in the 493–553 °K temperature and 480–730 MPa stress regimes, with an average activation energy between 121.6 and 148.2 kJ mol^−1^. These stresses are too high for A356, and the mechanisms would be related to plastic deformation rather than to creep.

De Luca et al. [29] investigated the creep resistance at 300 °C and 400 °C under stress ranges of 10 and 70 MPa and 5 and 30 MPa, respectively, using Mn and Mo additions in the Al-Zr-Sc-Er-Si-based alloy. The Al-0.08Zr-0.02Sc-0.01Er-0.10Si-0.25Mn-0.11Mo subjected to compressive creep in the aged condition indicated no degradation in the creep resistance at both testing temperatures. The researchers associated this behavior with the solid-solution strengthening and the formation of thermally stable α-Al(Mn,Mo)Si submicron precipitates (on the grain boundaries) and Al_3_(Zr,Sc,Er)(L1_2_)-nanoprecipitates (at the grain boundaries). Additional results revealed that the formation of fine equiaxed grains in the modified alloy (0.35 mm) and elongated grains (0.6 mm) in the base alloy, with inter-precipitate distances from ~10 μm to >100 μm, influenced the creep resistance. The deformation mechanisms were identified as diffusional creep at 400 °C for both alloys, and as dislocation glide for the modified alloy and dislocation climb for the base alloy at 300 °C. However, better creep resistance was obtained in the modified alloy due to the presence of α-Al(Mn,Mo)Si-precipitates that provided GB strengthening.

Certain findings of the early work on the third generation of aluminum alloys are worth summarizing:

1. Formation of submicron precipitates: Zr addition forms submicron precipitates (ternary Al-Si-Zr) in Al-Si-Mg-Cu casting alloys, which enhance creep resistance [18]. Zr, Sc, and Er also form Al_3_(Zr,Sc,Er)(L1_2_)-nanoprecipitates in aluminum [29], which improve creep resistance.
2. Modification of the Al-Mn-Fe-Si dispersoids for ductility: Cr addition [18] and Zr and Er, added singly or in combination [28], modify the Al-Fe-Si into Al-Cr-Mn-Fe-Si dispersoids and seem to have a role in improving ductility.
3. Thermally stable Al-Mn-Fe-Si dispersoids for creep resistance: Mn (<1 wt%) and Mo added in combination modify and improve the thermal stability of the Al-Fe-Si dispersoids, forming α-Al(Fe,Mn,Mo)Si dispersoids that improve creep resistance.

The exact role of the dispersoids/precipitates in creep behavior, e.g., the interaction of precipitates with dislocations, sub-grain and grain boundaries, and the evolution of the microstructure with time, was not clarified in these early studies with microstructural investigation.

### 3.2. Recent Studies on the Third-Generation Aluminum Alloys

Recent work on Al-Si-Mg-Cu alloys in 2023 focused mainly on identifying the creep mechanisms and the role of dispersoids in creep resistance. It was noted that the dispersoids impeded dislocation climb and/or restoration processes of sub-grain formation and sub-grain migration.

Li et al. [30] carried out compressive creep tests at 300 °C and 50 MPa for 100 h after T7 heat treatment in 356 alloy with individual and combined additions of V, Zr, and Mo. The study showed that the minimum creep rate decreased 67% and 50% for the alloys containing V and V-Zr-Mo, respectively, when they were compared with the base A356 alloy. The decrease was associated with the formation of large grain sizes, lower fraction of DFZ, and more rounded dispersoids. They also reported that the A356 alloy with V, Zr, and Mo addition exhibited the highest strengthening effect at 300 °C, with a 22% improvement compared with the A356 alloy due to a high-volume fraction of thermally stable (Al,Si)_3_(Ti,V,Zr) and α -Al(Mn,Mo,V,Fe)Si dispersoids. The stress used was, however, higher than that observed during diesel engine operation.

Qian et al. [31] studied a strengthening method based on multi-element additions and on the correlation between creep resistance and microstructure in the AA6111 (Al-0.95Mg-1.0Si-0.8Cu-0.1Mn-0.15Fe-0.1Cr-0.1Zn-0.08Ti) alloy strengthened by synergistic Er and Zr addition. The results showed better creep resistance in the Er- and Zr-containing alloy (165 kJ/mol) than in the alloy containing only Er (160 kJ/mol). Microstructural investigation related this improvement with the formation and refinement of the coarse Al_3_Er inter-metallics to Al_3_(Er, Zr) nanoprecipitates, which were characterized by their thermal stability (29 and 34 nm before and after creep, respectively) and produced through the combination of a Zr-enriched outer shell and an Er-enriched inner core. The Al_3_(Er, Zr) particles prevented grain boundary migration and the movement of the dislocation rate during creep deformation. They claimed dislocation climb to be the main creep deformation rate-controlling mechanism in both alloy systems. Alloy 6111, however, is a wrought alloy, and the principles need to be further developed for cast alloy compositions. Selective reinforcement of sections of the cylinder head via a wrought alloy is also a possibility that can be investigated.

**Figure 6:**
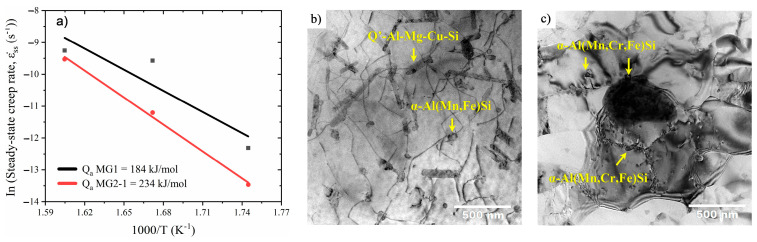
(**a**) Arrhenius plot of log of steady-state creep rate vs. the reciprocal of temperature at 28 MPa, (**b**) dislocation arrangements from interaction of the dislocations with α-Al(Mn,Fe)Si and Q’-Al-Mg-Cu-Si particles, and (**c**) interaction of α_MG2-1_-Al(Mn,Cr,Fe)Si particles with dislocations and sub-grain boundaries. Reproduced with permission from [32]. Copyright Elsevier, 2023.

Arriaga-Benitez and Pekguleryuz [32] carried out a systematic study on the creep behavior of Al-Si-Mg-Cu (Mn, Cr, Zr) alloys. Considering that threshold stress analysis does not reveal the creep micro-mechanisms and the specific role of the dispersoids in softening processes, the authors utilized in-depth electron microscopy analyses of creep-tested samples to theoretically assess the activation energy for creep and compare it to experimentally determined activation energies. The modified Al-7Si-0.3Mg-0.5Cu-0.15Mn-0.25Cr-015Zr (MG2-1) alloy activation energy of 234 kJ mol^−1^ indicated a higher apparent activation energy (Q_a_) of 50 kJ mol^−1^ than the base alloy Al-7Si-0.3Mg-0.5Cu (MG1) after the tensile creep tests at 300–350 °C and 28 MPa in the T7 condition, suggesting better creep resistance (Figure 6a). TEM microstructural observations in the base alloy showed that Q’ precipitates are cut by the dislocations and the nano-sized α-Al(Mn,Fe)Si, ~50 nm in diameter, pin the dislocations. Based on the TEM investigation and the apparent activation energy, the researchers attributed the creep-rate-governing mechanism to climb over non-coherent α-Al(Mn,Fe)Si (Figure 6b). On the other hand, the modified alloy presented dislocation/dispersoid interactions and sub-grain formation, wherein the creep mechanism was identified as sub-grain boundary migration with Zener pinning by coherent and ~5 nm α_MG2-1_-Al(Mn,Cr,Fe)Si dispersoids (Figure 6c). The theoretical assessment used in this research to determine the creep mechanisms was found to agree very well with the experimental results.

Some of the important findings of the recent work on the third generation of aluminum alloys can be summarized as follows:

1. Modification of the Al-Mn-Fe-Si dispersoids: V-Zr-Mo addition to A356 created a high-volume fraction of thermally stable (Al,Si)_3_(Ti,V,Zr) and α-Al(Mn,Mo,V,Fe)Si dispersoids that decreased the creep rate by 50% [30].
2. Formation of submicron precipitates: Er and Zr addition to wrought alloy 6111 generated Al_3_(Er,Zr) nanoprecipitates (~30 nm) that prevented grain boundary migration and dislocation motion during creep. A very important discovery was the chemical inhomogeneity of the precipitates with a Zr-enriched outer shell and an Er-enriched inner core [31].
3. The role of Cr-modified Al(Mn,Cr,Fe)Si dispersoids in creep resistance was determined. Coherent ~5 nm dispersoids produced Zener pinning of sub-grain boundaries, slowing softening processes during creep [32].

The remaining issues are still related to determining the exact creep-strengthening mechanisms in the alloys, comparison of alloys (creep rates) under identical creep conditions (of the diesel engine), and the optimization of the alloys for castability and manufacturability.

## 4. New Approaches Enabling the Elevated-Temperature Use of Aluminum

The first three generations of alloys for diesel engine applications were based on Al-Si-Mg-Cu systems with varying Cu or Mg content or with Ni or transition/peritectic element additions. Recent work on developing aluminum for elevated-temperature applications investigated different alloy systems (mostly Al-Cu alloys) and nanostructured and/or Al composites. The use of coatings, optimization of the fabrication process, and the development of statistical algorithms are also studies that can be enablers of aluminum use for the diesel engine. These material advances may promote creep strength by introducing grain boundary reinforcement, reduction of the processing defects, and optimizing processing techniques (e.g., heat treatment). Their use in the diesel engine is not straightforward, but these studies can pave the path to the development of new diesel engine aluminum materials and processes.

### 4.1. Al-Cu-Based Alloys

Al-Cu-based alloys have lower castability than Al-Si-based alloys, but can achieve higher strengths, especially through age hardening with the θ-Al_2_Cu precipitation. Al-Si casting alloys have over the years replaced Al-Cu alloys due to improved fluidity and castability imparted by the Si addition. Studies on Al-Cu alloys have been carried out since 2017 on the elevated-temperature strength and creep resistance of these alloy systems; however, the studies did not consider castability in conventional casting techniques.

Awe et al. [33] developed a high-temperature Al-27Cu-5Si cast alloy with 0–1.5 wt% additions of Ni through the high-cooling-rate casting technique. Tensile tests performed at 300 °C revealed that yield strength and ultimate tensile strength increased from 96 MPa to 267 MPa and from 208 MPa to 272 MPa, respectively, with increasing Ni content, but elongation decreased from 12.3% to 4%. On the other hand, yield strength and ultimate tensile strength at 400 °C for the 1.5% Ni alloy decreased by 70% and 62%, respectively, but the elongation increased from 4% to 7%. Thermodynamic calculations and SEM-EDS analysis concluded that the enhanced tensile properties at elevated temperatures in the Ni-containing alloys are attributed to a change in the solidification profile in the base alloy by Ni. A thermally stable Ni-rich phase (Al7Cu4Ni) is produced that forms ahead of the eutectic front at a temperature greater than the eutectic temperature. Due to the high Cu content, its castability and microstructural refinement required rapid solidification, and Ni together with Cu seems to have created ductility problems.

Gao et al. [34] studied the co-stabilization of θ′-Al_2_Cu and Al_3_Sc precipitates in Al-1.08 at.%Cu alloys, including 0.18 at.%Sc, 0.18 at.%Sc with 0.05 at.%Si, and 0.18 at.%Sc with 0.05 at.%Zr additions after thermal exposure at 300 °C on artificial aged samples. The results showed that Sc-Si and Sc-Zr microalloying enhanced the thermal stability of θ′-Al_2_Cu precipitates. However, an increase in the Si addition accelerated the growth of Al_3_Sc particles, leading to limited optimization on high-temperature resistance. Therefore, the combined Sc-Zr microalloying showed the most desirable effect on the thermal stabilization of the dual precipitates at 300 °C. Experimental evidence of atom probe tomography associated the θ′-Al_2_Cu thermal stabilization with a reduction in interfacial energy caused by solute segregation at the θ′-Al_2_Cu/matrix interface. Similarly, Al_3_Sc thermal stabilization was related to the formation of a Zr-rich outer shell that encapsulated the inner Sc-rich core and served as a diffusion barrier to limit the solute exchange across the Al_3_Sc-based particles and to restrain their growth during thermal exposure at 300 °C. The idea of surface segregation in dispersoids and precipitates seems to open an interesting future direction in dispersoid strengthening of aluminum alloys.

Li et al. [35] performed compressive creep deformation tests at 300 °C for 100 h and 50 MPa on Al-Cu 224 cast alloys with micro-level transition metals (TMs) of Zr, V, and Sc to evaluate the thermal stability of θ″/θ′-Al_2_Cu precipitates and L1_2_-Al_3_M dispersoids. Creep resistance under the T7A condition indicated that the minimum creep rate with Sc and Zr additions obtained the lowest value of 2.8 × 10^−9^ s^−1^ when compared with the other alloys, wherein the base alloy obtained the highest minimum creep value of 2.0 × 10^−7^ s^−1^. Microstructural investigation indicated that this behavior is due to the presence of a high amount of Al_3_(Sc, Zr) dispersoids in combination with finer θ′ precipitates that inhibited dislocation movement, resulting in a small creep strain (0.007 after 100 h). Researchers associated the low degree of coarsening of θ′-Al_2_Cu with the segregation of TMs at the θ′ interface, reducing its interfacial energy and acting as a diffusion barrier for Cu atoms. Similar to the study of Gao et al. [34], this study [35] also underlines the importance of surface segregation in precipitates and can be an important design tool for second-phase strengthening, as well as for aging heat treatment.

Bahl et al. [36] evaluated the effect of θ-Al_2_Cu precipitates at the grain boundaries on Al-Cu-Mn-Zr (ACMZ) alloys with 6 wt% Cu and 9 wt% Cu, with tensile and compressive creep tests performed at 300 °C. Microstructural analysis showed that θ-precipitates increased their volume fraction from ~0.7% in 6Cu to ~6% in 9Cu at the grain boundaries. However, tensile creep indicated that the alloy with 9Cu failed in creep faster than 6Cu at stresses above 20 MPa, while deformation rates in the stress range of 115–110 MPa were not affected in compressive creep. Microstructures after creep testing related this behavior with a higher formation of cavities on grain boundaries promoted by large-sized intergranular θ-precipitates in 9Cu during tensile creep. This study, however, used stress levels higher than the service stresses of the diesel engine.

These recent studies on the Al-Cu system reveal that the Al-Cu-Ni system highlights the need for the addition of low-level elements to improve ductility and the thermal stability of the aging precipitates or to create thermally stable precipitates. The combined addition of Zr and Sc, and especially the presence of Zr, emerge as important alloy design tools for the future development of the Al-Cu system for elevated-temperature use.

### 4.2. Al Matrix Composites

Aluminum-based matrix composites (AMCs) combine an Al matrix with reinforcing phases (borides, carbides, oxides, nitrides, and their combinations) in the form of particles, whiskers, or fibers, leading to the development of lightweight and heat-resistant diesel engine components [37,38,39]. Some recent studies on this subject are presented below.

Ji et al. [40] evaluated the creep performance on Al-12Si alloy reinforced with borate whiskers (Al_18_B_4_O_33_) and unreinforced Al-12Si alloy at 250–400 °C and 50–230 MPa. Creep resistance in the reinforced alloy was improved by three orders of magnitude, compared with the unreinforced alloy. TEM microstructural investigations after the creep test indicated that the dislocations accumulated near the reinforced whiskers, which restricted their motion; in addition, the whiskers hindered the grain growth, leading to a more refined structure. However, due to the strain mismatch between the whiskers and the Al matrix, a stress concentration zone was formed around the whisker, causing its fracture. The creep fracture surfaces at 300, 350, and 400 °C showed that the fracture mechanism was related to reinforced whiskers, interfacial debonding between the whisker and matrix, and interfacial debonding and whisker fracture, respectively.

Zhao et al. [41] studied the creep behavior of the in situ (ZrB_2_ + Al_2_O_3_)/AA6016 composites prepared by the electromagnetic induction heating furnace. The AMCs in the T6 condition (545 °C for 2 h and 180 °C for 3 h) tested at 300 °C and 70 MPa showed creep lives of 9.1 and 11.2 h, respectively, when the particle content reached 1 and 3 vol.%. However, a reduction of 5.5 h with 5 vol.% was seen due to the stress concentration, which is caused by the presence of large particle clusters. Microstructural observations indicated that the introduction of ZrB_2_ (61 nm) and Al_2_O_3_ (49 nm) nanoparticles inhibited grain growth (68 μm) when compared with the 6016 alloy. In addition, θ and β phases decreased their diameters to 17 and 168 nm from 26 and 201 nm in the composite, inhibiting the dislocation movement, resulting in a higher creep resistance.

Huang et al. [42] investigated the synergetic effect of 1.3 wt% Al_2_O_3_ and 4.4 wt% ZrB_2_ in the 7075-aluminum alloy to determine the creep resistance after the hot rolling deformation. The homogenized (743 K for 24 h) (Al_2_O_3_ + ZrB_2_)/7075 Al composites subjected to 523, 543, and 563 K and 60, 70, and 80 MPa showed an apparent activation energy of ~370 kJ mol^−1^, indicating the stability and efficiency of the nanoparticles at high temperatures. Particle characterization revealed that the average grain size in the reinforced composite was the smallest, with 4.07 μm, compared to the 7055 Al alloy (6.31 μm) and ZrB_2_/7055 Al composites (4.42 μm). The authors associated this behavior with the inhibition of the recrystallization process due to the nanoparticles (Al_2_O_3_ and ZrB_2_) and precipitates (Cu, Mg, and Zn), wherein a high concentration of precipitates was easy to obtain due to their nucleation at the interface between the nanoparticles and the Al matrix.

Abd-Elaziem et al. [43] reviewed the effect of metal-matrix nanocomposites on the creep behavior of aluminum alloys. The literature studied indicated that hard, non-shearable, incoherent nanoparticles in the Al matrix may increase the creep resistance by reducing load transfer and dislocation activities at the matrix reinforcement interfaces. This assumption was made because the creep deformation of Al and its alloys is mostly caused by dislocation climb or gliding processes. Researchers also pointed out that smaller particle sizes may have major implications on the creep resistance due to their smaller spacing among each other. As a result, stiffer reinforcement can carry more stress load, allowing for higher applied stresses.

Studies have revealed that although the properties of Al alloys are improved with the addition of reinforcing second phases, factors such as porosity from composite processing, brittleness in the reinforcement material, and poor wettability in terms of bonding between the matrix and reinforcement may result in a reduction of the creep resistance with increasing temperature [44,45]. Therefore, a comprehensive review of the selection of proper reinforcement materials and methods to produce a homogenous distribution of the reinforcements in the Al matrix is needed to achieve the desired creep properties of the composites. Systematic studies on rate-governing creep mechanisms and activation energies would also be good contributions to the understanding of the roles of the reinforcements in creep behavior.

### 4.3. Additive Manufacturing

The additive manufacturing (AM) process provides a tool to create complex geometries, leading to the development of cylinder head and engine block designs [46]. In Al alloy design, AM may offer better creep performance over conventional manufacturing processes due to the formation of refined precipitates, control of particle distribution, and the ability to create high-volume fractions of thermally stable phases [47]. However, defects such as inhomogeneous microstructure, hot cracking, porosity, anisotropy, poor surface quality, and residual stresses during AM processing lead to deficient material properties [48,49]. Some recent studies that have contributed to this trend are presented below.

Michi et al. [50] investigated a creep-resistant, additively manufactured Al-Ce-Ni-Mn alloy in the 300–400 °C temperature range. The results indicated that the Al-10.5Ce-3.1Ni-1.2Mn alloy showed superior creep resistance to Al-12.5Ce, Al-6.4Cu-0.19Mn-0.13Zr, and Al-0.10Sc-0.12Er cast alloys. This behavior was associated with a higher volume fraction (35%) of submicron Al_11_Ce_3_ and Al_3_Ni precipitates, which are thermally stable up to ∼400 °C, that controlled the dislocation climb and acted as barriers to grain boundary migration. A low creep ductility in the alloy was observed due to the appearance of voids at the melt pool boundaries (MPBs) during AM processing.

Glerum et al. [51] investigated the creep properties and microstructure evolution at 260–300 °C of AlSi10Mg manufactured via laser powder-bed fusion. The Al-10Si-0.6Mg alloy subjected to compressive creep in the aged condition indicated an apparent activation energy equal to 256 kJ mol^−1^ when tested at 200–320 °C and 45 MPa. The researchers compared the creep response to cast alloys strengthened via load transfer (Al-12.5Ce, Al-10Ce-5Ni, and Al6.9Ce-9.3Mg). Microstructural observations revealed that the Si phase in the LPBF alloy was much finer than the eutectic Al_11_Ce_3_ phase in the cast alloys due to the rapid solidification rate from the LPBF process.

Rakhmonov et al. [52] examined the laser-melted Al-3.6Mn-2.0Fe-1.8Si-0.9Zr (wt%) alloy with outstanding creep resistance due to the formation of α-Al(Fe,Mn)Si precipitates. Compressive creep results at 300 °C and 50–80 MPa on the aged alloy (350 °C for 8 h) showed lower stress exponents with better creep resistance when the alloy was compared with creep-resistant cast and AM Al alloys. This behavior was associated with the formation of submicron, thermally stable α-Al(Fe,Mn)Si precipitates with a high-volume fraction obtained from the rapid solidification rate of the LPBF process. The study revealed that α-Al(Fe,Mn)Si precipitates were responsible for refining the α-Al grains, preventing cracking during LPBF processing, and inhibiting diffusion-controlled dislocation climb, generating an improvement in the creep resistance.

The key finding in studies on AM aluminum alloys is the observation of fine dispersoids/precipitates obtained because of the high solidification rates in LPBF. Submicron Al_11_Ce_3_ and Al_3_Ni precipitates, fine eutectic Si phase, or α-Al(Fe,Mn)Si dispersoids were able to provide creep strengthening at temperatures in excess of 300 °C.

### 4.4. Thermal Barrier Coating

Thermal barrier coatings (TBCs) have the potential to improve the engine power and efficiency because they can be used to insulate the engine block and cylinder head (i.e., the inter-valve bridge area) against the thermal effect of the combustion gases responsible for producing creep deformation [53]. However, their use has been limited due to defects, such as porosity, cracking, residual stress, oxidation, thermal shock, and microstructure, which may influence their performance [53,54]. In addition, factors such as high surface roughness (increases heat transfer and slows down combustion) and translucency to thermal radiation may affect their effectiveness [55,56]. Recent studies in this field are presented below.

Wang et al. [57] investigated a high-entropy Al_0.6_CoCrFeNiTi alloy coating on the aluminum 4032 alloy substrate. The coating, formed via plasma spraying, showed lower thermal conductivity (3.00 W/mK at 100 °C) and higher thermal stability (up to 700 °C) than high-velocity oxygen-fuel-spraying. This indicated that the operating surface temperature of the piston crown could increase by 13 °C because the coating provided a temperature reduction of 19 °C. The researchers associated this behavior with the refinement of grains and disordered BCC structure produced from the combination of a high cooling rate and sluggish diffusion in Al_0.6_CoCrFeNiTi. The study also revealed that defects such as porosity and oxide inclusions influenced thermal insulation properties.

Vengatesan et al. [58] studied the influence of alloying elements on the performance of an Al-Si engine piston coated with 350 mm of MgZrO_3_ and 150 mm of NiCrAl. It was reported that a high content of alloying elements, including Cu and Ni, required more time to heat the Al-Si alloy substrate, altering the thermal conductivity of the piston exterior. As a result, an increase in the temperature in the piston surface was seen, leading to an improvement of 55% in the surface temperature when compared to the aluminum alloy. Similarly, an increase in the coating thickness led to higher values in temperature than those in the uncoated piston. However, the authors associated the best thermal behavior with coatings containing holes, showing improvements of about 8–10% compared to pistons without holes and 32–47% compared to uncoated pistons.

M. Cerit and M. Coban conducted simulations on the temperature and stress distributions of an Al-Si-Cu-Mg-Cr alloy piston, plasma-sprayed with a magnesia-stabilized zirconia (MgZrO_3_) thermal barrier coating, an intermediate layer of NiCrAl bond coat between the piston surface, and a ceramic topcoat [59]. They obtained a 9–20% reduction in the temperature and 33–57% reduction in the residual stresses of the piston’s metal surface depending on the coating thickness.

The recent studies on TBC were all conducted on the pistons and not on the engine block: MgZrO_3_ with an intermediate layer of NiCrAl produced temperature reductions of a minimum of ~10–20%, and the high-entropy Al_0.6_CoCrFeNiTi coating produced a reduction of ~20 °C (which can translate into a 7% reduction in temperature), providing a small improvement in creep behavior. The use of TBCs in the engine block can be a design element, as it can decrease the metal surface temperature and, consequently, the creep temperature in the engine block, provided that good-quality, thick coatings can be achieved.

### 4.5. Nanostructured Aluminum Alloy

Nanostructured (NS) aluminum alloys produced from mechanical alloying, severe plastic deformation, and electrodeposition are considered as potential candidates to be used in lightweighting applications at elevated temperatures due to the combined effect of ultrafine grains and nanoparticles [60,61]. Several types of NS aluminum alloys have been developed [62]. These materials are not for cast parts. Although studies [63,64] on the diesel engine piston have shown suitable strength at elevated temperatures, no research has been conducted on engine blocks and cylinder heads. This may be due to low thermal stability in the diesel engine operating condition as well as the difficulty of producing a complex shape, such as the engine block, with these materials. There is an effort in this field with respect to thermal instability and grain size stabilization through thermodynamic stabilization and kinetic stabilization that is of interest to diesel engine applications [65,66]. The implementation of these materials in a cast diesel cylinder head is not straightforward. However, a future direction can be the use of these materials for selective strengthening of certain parts of the cylinder head. Recent studies in this area are presented below.

Markushev et al. [67] investigated the role of dispersed particles in nanocrystalline Al-Zn-Mg-Cu-Sc-Mn alloys under high-pressure torsion (HPT). The results indicated the absence of recrystallization in HPT samples after the pre-age condition (170 °C for 10 h) due to the high density of thermally stable transition metal aluminides. In addition, microstructural observations revealed that the suppression of the Al matrix nano-structuring was associated with the homogeneity of plastic flow and prevention of dislocation rearrangements. In contrast, in the over-aged condition (250 for 5 h), the main strengthening phases increased their size and decreased their density, resulting in a reduction in hardness. A better understanding of the principles of optimization was obtained to develop stable nanostructures during service conditions.

Bobruk et al. [68] studied the Al 2024 alloy with ultrafine grains produced by HPT in combination with nanoscale precipitates of Al_2_CuMg. Tensile mechanical tests at 190, 240, and 270 °C under a strain rate of 10^−3^ s^−1^ demonstrated elongations of 130, 280, and 400%, respectively. Similarly, the microhardness was only slightly reduced, indicating the thermal stability of the alloy with increasing temperature. The super-plasticity behavior was associated with a uniform distribution of nanosized precipitates of Al_2_CuMg phase and grain boundary segregation that suppressed the recovery and recrystallization process. TEM analysis also indicated that the grain size of 0.1 μm was preserved in the 120–270 °C temperature range, with no structure degradation.

Galano et al. [69] reviewed the creep and deformation mechanisms at elevated temperatures in Al-based nano-quasi-crystalline alloys that had quasi-crystalline phases with high Al concentrations, such as Al_78_Mn_22_, Al_86_V_14_, and Al_84.6_Cr_15.4_. They pointed out that the thermal stability of the nano-quasi-crystalline-based alloys is mainly related to the stability of the metastable icosahedral phase, wherein phase and interface stabilization in the α-Al matrix should be maintained to preserve the microstructure, without considerable changes at elevated temperatures. The study indicated that the nano-quasi-crystalline Al-Fe-Cr-based alloys have improved mechanical properties at elevated temperatures when compared with standard Al alloys. In terms of creep behavior, the Al-Fe-Cr-Ta alloy showed an apparent activation energy of 440 kJ mol^−1^ in the temperature range of 573 K and 623 K due to the formation of fine particles with small inter-particle distances that controlled the dislocation motion.

Future strategies in the NS Al alloy design, such as the selection of stabilizing alloying elements, size, volume fraction and distribution of second-phase particles, and microstructural evolution, need further investigation. The incorporation of thermally stable nanostructured aluminum inserts in the cylinder head will be a viable engineering route in the development of aluminum diesel engines.

### 4.6. Machine Learning Design for Al Alloys

Machine learning (ML) has become a useful approach in Al alloy design using complex and high-dimensional datasets to predict the mechanical properties at high temperatures [70,71]. In creep-oriented alloy design, ML has been used to predict the primary parameters associated with creep behavior (lifetime, creep rate, creep strain, and creep curves) in different alloy systems [72,73]. However, its study in Al alloys remains untouched. Among the main factors is the limited data available, wherein alloy composition, microstructure, and processing conditions are included in the datasets for the resulting creep behavior [74]. The ML studies on aluminum have focused on optimizing heat treatment or fatigue properties, as discussed below.

Jiang et al. [75] designed a three-stage solution aging process using data transfer learning of 1053 data points on AA7xx series high-strength aluminum alloys. The prediction results were carried out on the Al-8.9Zn-2.29Mg-1.76Cu-0.14Cr-0.1Mn-0.1Zr-0.06Ti alloy previously determined by the authors using the machine learning method [76]. The UTS and elongation in the T66R process (450 °C for 1 h + 470 °C for 1 h + 480 °C for 0.5 h + 65 °C for 42 h + 100 °C for 16 h + 135 °C for 4 h) showed higher values than those of common T6-treated samples (470 °C for 2 h + 120 °C for 24 h). Experimental verification of the alloy revealed that UTS and elongation increased from 715 to 767 MPa and from 8.4 to 13.4%, respectively. Microstructural investigation associated this behavior with the reduction of the amount of micron-scale insoluble phases, wherein finer and spherical dispersed nanoprecipitates increased their density by more than 50%.

Lian et al. [77] employed empirical formulae and data-driven models to predict the fatigue life of seven different series of Al alloys, with the purpose of handling small datasets. The transfer learning used in this work included fatigue strength and S-N curves obtained from literature, and the construction of two models, fatigue strength and S-N curves. As a result, quantitative relationships in the fatigue life based on the S-N curve model were obtained.

There is current research geared toward designing Al-Si-based diesel engine alloys by the authors using transfer learning models on datasets from aluminum and magnesium alloys and superalloys [78]. Ongoing work shows that creep data on aluminum casting alloys are limited. Transfer learning methods can be applied to magnesium, steel, or superalloy datasets, or parametric analyses can be performed to create new data. ML strategies, such as transfer learning, feature engineering, and physics-informed models, can be used in the future to address the data-related limitations.

### 4.7. The Manufacturing of the Aluminum Diesel Engine

The manufacturing of the diesel engine basically relies on casting processes. However, incorporating material solutions that are not developed for casting into the production of an aluminum diesel engine block is not straightforward. In this section, the conventional casting processes of aluminum alloys are summarized, and the selective reinforcement of the aluminum engine block is discussed.

#### 4.7.1. Casting Processes for Aluminum Engine Block [79,80]

Commercial aluminum casting alloys are developed and optimized for specific casting processes (Table 1). Commonly, thin-walled castings, such as the transmission case, can be cast via the high-cooling-rate HPDC (high-pressure diecasting) process, which generates supersaturated solid solution and refined microstructures that lead to high tensile properties without heat treatment. In fact, heat treatment cannot be applied to HPDC, unless the part is hot-isostatic-pressed to remove the air entrapped during turbulent flow into the die, which leads to blistering problems during heating. HPDC is not the most suitable technique for thicker wall castings, such as the engine block, due to high amounts of air entrapment and lower cooling rates obtained in thick sections. The engine block is preferentially cast using the permanent mold (PM) casting (or modified sand casting) with and without pressure assistance, e.g., gravity casting (GC) or low-pressure casting (LPC) techniques. To improve the casting soundness and properties of GC and LPC, the melt can be treated (degassed, grain refined, and chemically modified) [80].

Al-Si-based Alloys: Modifications of the A356 alloy constitute the bulk of the studies [18,19,20,21,22,23,24,25,26,27,28] of the first-, second-, and third-generation alloys. Cu and Ni additions, as well as low-level additions, can affect the castability and ductility of A356. However, no systematic study on the castability (hot-tearing tendency, porosity, and fluidity) of these alloys has been conducted.

Al-Cu-based Alloys: The alloys investigated in this system are highly experimental at this stage, sometimes containing high (9–27 wt%) Cu content. The value of these studies lies in the insight they provide into the alloy features that enhance creep resistance. The exception is the study of the Al-Cu 224 cast alloy [35], which is the modification (microalloying) of a commercial PM casting alloy.

#### 4.7.2. Selective Reinforcement in the Diesel Engine

Selective reinforcement has been used in automotive engine applications in the past. In situ selectively reinforced (SF) pistons using liquid metal infiltration of ceramic fiber preforms was introduced by Toyota diesel engines in 1983. Subsequent uses of SF using aluminum matrix composites (AMCs) were piston cylinder bores by Porsche (Stuttgart, Germany) (Boxster, 911 Carrera) and since 1990 by Honda (Tokyo, Japan) (Prelude SI, Accord, V6 engine of Acura NSX, and S2000 Roadster). The design with selective reinforcement was successfully used by BMW on their creep-resistant magnesium AJ62 alloy engine block in all models between 2004 and 2015, using a cast aluminum alloy insert in the piston area to provide improved creep resistance in this region [81].

The inter-valve area of the diesel engine is positioned to benefit significantly from selective creep reinforcement using many of the materials discussed in Section 4.1–Section 4.3.

Al-Cu alloys [33,34,35,36], metal-matrix composites [37,38,39,40,41,42,43,44,45], and additively manufactured aluminum [46,47,48,49,50,51,52] can be used as selective reinforcement materials. Nano-structured aluminum [60,61,62,63,64,65,66,67,68,69] can be used as inserts to improve the creep resistance of the aluminum diesel engine in the inter-valve zone if the thermal stability can be improved. An integrated technology similar to the one that has been proposed for the integration of AM parts into subtractive production processes [82] can be used by employing robot arms to move inserts, molds, and molten alloy to produce the component.

## 5. Conclusions and Future Directions

The review of the literature and research on the development of creep-resistant aluminum alloys intended for diesel engine applications showed that diesel engines will continue to be employed in heavy vehicles due to the limitations of battery technology and charging infrastructure of electric vehicles. High thermal gradients occurring during diesel engine operation render creep resistance a key requirement for the diesel engine cylinder head and engine block. The review also showed that there is still much work to be carried out before aluminum diesel engines can become commercially viable. The following points will pave the path to the effective development of this important application.

Effective Dispersoids: Research shows that dispersion strengthening is an effective way to control creep deformation, achieved by the addition of transition metals to Al-Si- or Al-Cu-based alloys. The dispersoids slow softening processes, such as dislocation climb, sub-grain formation, and sub-grain migration, minimizing dynamic recovery. The extent of dispersoids’ effectiveness depends on their thermal stability, size, and coherency.

A direct comparison of the experimental alloys investigated in the studies reviewed is difficult, as the creep stresses and temperatures differ. There is also a difference between tensile and compressive creep rates, especially when cast samples have porosity, inclusions, or very brittle phases that cause early failure in tensile creep. The minimum creep rate can be lowered by an order of magnitude. Furthermore, experimental alloys are usually not optimized for casting, and the tensile properties may be lower than the values obtained on sound castings. A partial comparison is provided in Table 2, which presents steady-state (minimum) creep rates and second phases that provide creep resistance.

In Al-Si-Cu-Mg alloys, modifications of α-Al(Mn,Fe)Si with low additions of Mo, Mn, and Cr have a beneficial role by increasing the coherency, thermal stability, and the coherency of the dispersoids. Though the findings can still be considered a work in progress, the efficiency of the modified dispersoids can be ranked in descending order of effectiveness as:

α-Al(Fe,Mo)Si > α-Al(Mn,Cr,Fe)Si > α-Al(Mn,Mo,Fe)Si

Al-Cu-based alloys show lower minimum creep rates than the Al-Si-based alloys. However, these alloys are known to have lower castability and can have ductility and corrosion problems. In both alloy systems, the combined addition of Zr and Sc generates the Al3(Sc,Zr) phase, which seems to lead to the lowest creep rates.

Future studies should emphasize creep tests that use the conditions of the diesel engine service temperatures and stresses. Compressive creep is more representative of the inter-valve creep conditions, but it may mask the inherent problems of the alloys, such as their tendency to crack formation. Tensile creep tests underestimate the alloy’s creep resistance if the samples have casting defects. Alloy comparisons must be made either on compressive or tensile creep or both. The improvement of the ductility and castability of the Al-Cu alloys is also a topic of interest for the future.

Alternative ways of improving creep resistance or elevated-temperate performance in aluminum: Alternative ways (other than casting alloys) were also reviewed in this paper. Composites can develop creep resistance by inhibiting recrystallization but can suffer from interface debonding. An interesting direction in the development of aluminum diesel engine alloy is through additive manufacturing (AM). The development of submicron dispersoids is very effective in improving creep resistance in AM aluminum alloys. The processing of these alloys does not lead to effective manufacturability of the diesel engine block but selective reinforcement of the block in the inter-valve region using inserts of these materials. This is an equally important topic to investigate in the future. Nano-structuring is seen to be challenged by thermal instability, requiring further research in stabilization. Their incorporation into the aluminum diesel engine as selective reinforcers would be a viable future direction. Thermal coatings can reduce creep deformation by limiting the temperature increase in the alloy.

The future trends in heat-resistant aluminum alloys involve their process optimization, alloy design by dispersoid design and/or via machine learning, incorporation of inserts of AM and AMCs, and advanced coating technologies to obtain Al alloy components with longer durability, higher efficiency, and better performance. The development and deployment of these materials and processes in diesel engines need to be accelerated to improve sustainability in heavy vehicles. The research can benefit from funding of multi-partner precompetitive research projects that combine multiple end-users, multiple alloys, alloy suppliers, and manufacturing into a systematic study with a focus on application.

## Figures and Tables

**Table 1:** Casting processes for commercial aluminum alloys.

Alloy	Casting Process	Alloy System	Applications
Al-Si-based alloys			
356, 357	Sand, PM, vacuum-assisted diecasting	Hypoeutectic Al-Si with 0.35 wt% Mg	Aircraft and aerospace, wheels, pillars, sub-frame
319	PM	Hypoeutectic Al-Si with 4% Cu	Cylinder heads, manifolds, engine blocks, internal engine part
380	HPDC		Wheels, transmission case
355, 332	PM	Hypoeutectic Al-Si with Cu and Mg	Pistons and engine block (gas or diesel)
390	PM, HPDC	Hypereutectic Al-Si with Cu and Mg	Wear applications, engine block without iron sleeves
Al-Cu-based alloys			
201	Sand cast, PM	Al-Cu with Ag	Aircraft engines
242	PM	Al-Cu with Ni and Mg	Diesel engine pistons, air-cooled cylinder heads for aircraft engines
224	PM	Al-Cu	Catalytic converters, heat shields

**Table 2:** Creep parameters of experimental aluminum alloys for the diesel engine.

Alloy (Compositions in Weight Percentage)	Min. Creep Rate (s^−1^)		Creep Enhancers	Ref.
	Tensile	Compressive		
Al-Si-based alloys creep-tested at 300 °C and 30 MPa				
A356 + 0.5Cu	4.5 × 10^−6^	2 × 10^−7^	50 nm noncoherent α-Al(Mn,Fe)Si	[18,32]
A356 + 0.5Cu + 0.2Mn + 0.2Cr + 0.2Zr	1.4 × 10^−6^	-	5 nm coherent α-Al(Mn,Cr,Fe)Si	[32]
A356 + 0.5Cu + 0.3Mo	1.0 × 10^−6^	-	α-Al(Fe,Mo)Si	[25]
A356 + 0.5Cu + 0.3Mo + 0.2Mn	2.1 × 10^−6^		α-Al(Mn,Mo,Fe)Si	[27]
Al-0.1Si-0.1Mn-0.1Mo-0.08Zr-0.01Er-0.02Sc	-	2 × 10^−7^	Al_3_(Zr,Sc,Er), submicron α-Al(Mn,Mo)Si	[29]
Al-Cu-based alloys creep-tested at 300 °C and 50 MPa				
Al-5Cu-(Si, Fe, Mg, Ti 0.1%ea)	-	2.2 × 10^−7^	θ′, θ″	[35]
Al-5Cu-0.18Zr-(Si, Fe, Mg, Ti 0.1ea)	-	2.8 × 10^−8^	θ′, θ″, Al_3_(Zr)	
Al-5Cu-0.25V-(Si, Fe, Mg, Ti 0.1ea)	-	8.4 × 10^−8^	θ′, θ″	
Al-5Cu-0.15Zr-0.13V-(Si, Fe, Mg, Ti 0.1ea)	-	1.3 × 10^−8^	θ′, θ″	
Al-5Cu-0.15Zr-0.16Sc-(Si, Fe, Mg, Ti 0.1ea)	-	5.5 × 10^−9^	θ′, θ″, Al_3_(Sc,Zr)	
Al-6Cu-0.4Mn-0.16Zr-0.08Si	7.0 × 10^−8^	6.0 × 10^−8^	θ′, θ	[36]
Al-8.5Cu-0.4Mn-0.16Zr-0.07Si	2.0 × 10^−8^	6.0 × 10^−9^	θ′, θ	

## Data Availability

The original contributions presented in the study are included in the article, further inquiries can be directed to the corresponding author.
